# A Rare Case of Concomitant Duodenal and Gallbladder Perforation in a Pediatric Patient

**DOI:** 10.7759/cureus.82838

**Published:** 2025-04-23

**Authors:** Soufiane Essamoud, Mohamed Lahlou, Achraf Benmazhar, Rania Bennani, Yannis Ennachachibi, Mouna Khmou, Basma Elkhannoussi, Hicham Zerhouni

**Affiliations:** 1 Department of Pediatric Surgery, Mohammed VI University of Sciences and Health, Casablanca, MAR; 2 Department of Pediatric Surgery, Mohammed VI International University Hospital of Bouskoura, Bouskoura, MAR; 3 Department of Anatomical Pathology, Mohammed VI University of Sciences and Health, Casablanca, MAR; 4 Department of Anatomical Pathology, Cheikh Khalifa International University Hospital of Casablanca, Casablanca, MAR; 5 Faculty of Medicine and Pharmacy of Rabat, Mohammed V University, Rabat, MAR; 6 Department of Anatomical Pathology, National Institute of Oncology of Rabat, Rabat, MAR

**Keywords:** duodenal perforation, gallbladder perforation, gastrointestinal complications, pediatric surgery, stress ulcers

## Abstract

Concomitant perforation of the duodenum and gallbladder is exceptionally rare in the pediatric population. These conditions pose significant diagnostic and therapeutic challenges, particularly in critically ill or sedated children.

We report the case of a five-year-old girl who was initially hospitalized for posterior fossa tumor resection and developed acute abdominal distension, anemia, and hematemesis in the postoperative period. Imaging was limited because of the hemodynamic instability. Emergency exploratory laparotomy revealed a large duodenal perforation and a concomitant gallbladder perforation. The surgical management included primary duodenal repair, cholecystectomy, and gastroduodenal bypass. Despite intensive supportive care, the patient succumbed to sepsis and multiorgan failure on postoperative day 2.

This case highlights the diagnostic difficulty of gastrointestinal perforations in sedated intensive care unit patients, the possible pathophysiological link between duodenal ulcers and adjacent gallbladder inflammation, and the urgency for surgical intervention.

Clinicians should maintain a high index of suspicion of stress-related gastrointestinal complications in critically ill children. Early surgical exploration may be life-saving when imaging is inconclusive and clinical deterioration is rapid.

## Introduction

Duodenal perforation is relatively uncommon in the pediatric population and often presents significant diagnostic challenges due to atypical symptoms and the risk of rapid deterioration if not promptly recognized and treated [1]. Similarly, isolated gallbladder perforation is also an uncommon finding in children [2]. Given the anatomical proximity of these organs, complications may arise [3]. Furthermore, while isolated cases have been documented, the concomitant occurrence of both is exceedingly rare, particularly in the pediatric setting [4]. This presents an even more complex clinical scenario that requires careful evaluation and management to ensure optimal outcomes. To date, only one similar case of dual perforation in a child has been reported in the medical literature [5].

This case report describes the clinical presentation, diagnostic limitations, surgical findings, and management of a five-year-old girl who developed concurrent duodenal and gallbladder perforations during a prolonged intensive care unit (ICU) stay following neurosurgical intervention. This report underscores the importance of clinical vigilance and timely surgical exploration in critically ill pediatric patients, especially those at risk of stress-related ulcers.

## Case presentation

Clinical presentation

**Figure 1:**
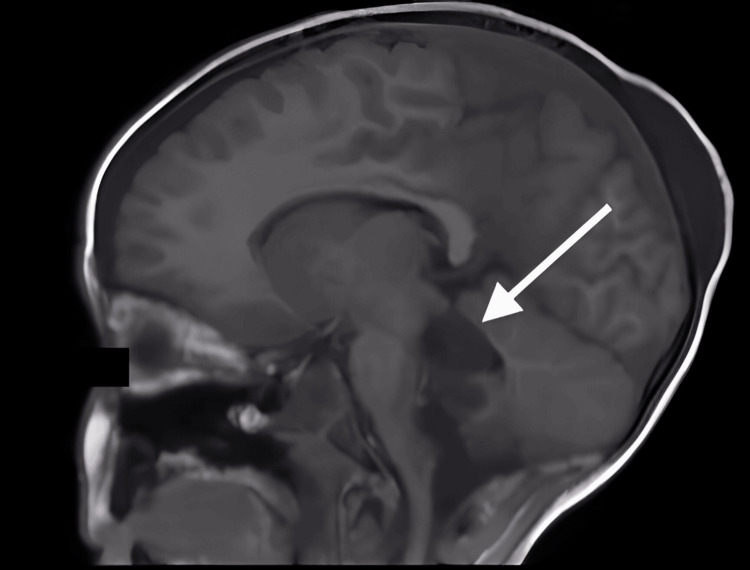
MRI image in the sagittal plane of the tumoral process in the posterior cerebral fossa

A five-year-old girl was admitted to the neurosurgery department with chronic vomiting, dizziness, and loss of equilibrium. She was incorrectly diagnosed with chronic gastritis and treated with proton pump inhibitors (PPIs) for three months, which unfortunately delayed the correct diagnosis and timely treatment of her underlying condition. MR imaging revealed a mass in the posterior cerebral fossa compressing the brainstem and cerebellum, leading to her symptoms and hydrocephalus (Figure 1). The patient underwent placement of a ventriculoperitoneal (VP) shunt as the primary surgical intervention. One month later, the patient underwent a craniotomy for tumor resection.

She was then admitted to the ICU for close monitoring and management of postoperative recovery. She was sedated and intubated for five days. Complications arose during her stay, with abdominal distension, pallor, an acute drop in hemoglobin from 8.4 g/dl to 3.4 g/dl overnight, and active hematemesis after stomach aspiration. C-reactive protein (CRP) level was 201.9 mg/l, procalcitonin level was >100 ng/ml, prothrombin level was 62%, and WBC = 20,900/mm^3^ (Table 1).

**Table 1:** Laboratory investigations according to the day of the laparotomy exploration

Parameter	One day before intervention	The day of intervention	Day 1 postoperative	Day 2 postoperative	Reference range
Hemoglobin (g/dl)	8.4	3.4	10.1	6.2	10.9-13.7
White blood count (WBC) (10^3^/mm^3^)	9.21	20.90	20.86	27.00	5-12
Platelets (10^3^/mm^3^)	215	101	104	76	200-400
Prothrombin (%)	57	62	28	45	70-100
C-reactive protein (CRP) (mg/ml)	-	201.9	30	-	<2.8
Procalcitonin (ng/ml)	-	>100	>100	-	<0.5

Diagnostic workup

**Figure 2:**
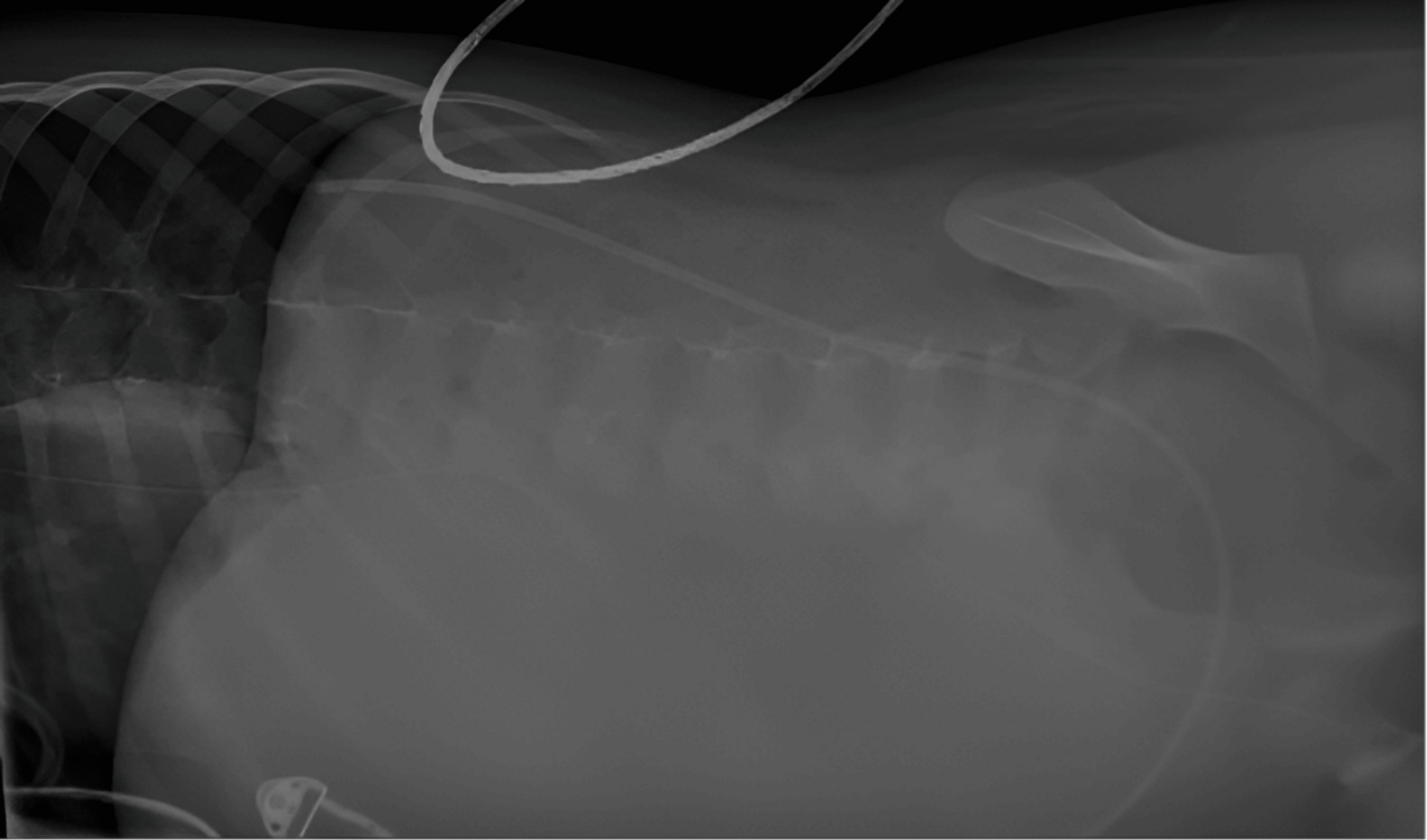
Lateral supine plain abdominal radiography. Abdominal air leakage is absent

A focused assessment with sonography in trauma (FAST) ultrasonography revealed abundant hyperechogenic fluid in the abdominal cavity, indicating possible intra-abdominal bleeding. The surgical team was alerted, an urgent lateral supine plain abdominal X-ray was performed to assess for any signs of perforation, and the results showed no evidence of air in the abdomen (Figure 2). No CT scan was performed because of hemodynamic instability and transportation risks to the radiology department.

Blood transfusion was initiated to stabilize her hemoglobin levels and improve her overall condition, along with the administration of noradrenaline as a vasoactive agent. Thus, the decision was made to surgically explore the abdomen to identify the source of bleeding.

Surgical intervention

**Figure 3:**
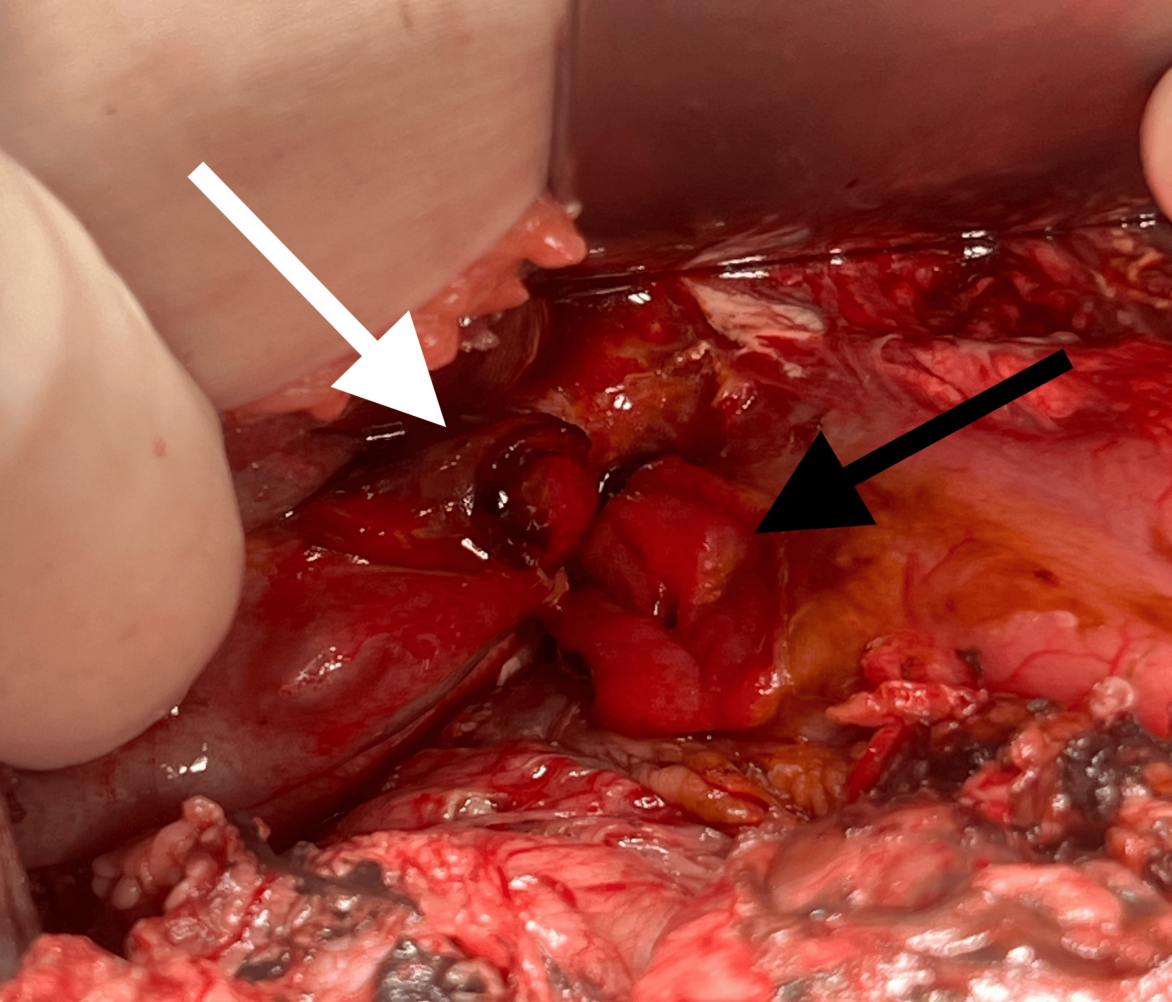
Perioperative findings: duodenal perforation (black arrow) and gallbladder perforation (white arrow)

After stabilization, the patient was placed in the operating room (OR). The preoperative diagnosis was gastric ulcer stress-induced perforation despite the lack of confirmation. An upper umbilical transverse abdominal incision was made. At the opening of the peritoneum, 2 liters of greenish fluid were aspirated, indicating the presence of bile and peritonitis. Thorough exploration of the abdomen revealed a perforation on the anterior wall of the first segment of the duodenum (4 cm in size) and a smaller adjacent gallbladder perforation (Figure 3) with bile leakage into the peritoneal cavity. The VP shunt was observed to be freely floating within the abdominal cavity, without any adhesion to the bowel, and was consequently ruled out as the cause of the perforations.

The surgical team proceeded to repair the duodenal perforation using a primary closure technique with interrupted 4-0 resorbable sutures, and cholecystectomy was performed to address the gallbladder perforation. Because of the high risk of duodenal leakage, gastrojejunal bypass was performed to divert the flow of gastric contents away from the repaired area, ensuring better healing and minimizing complications. The abdominal cavity was carefully irrigated with saline to remove any residual bile and debris, followed by closure of the abdominal wall after the placement of two different passive peritoneal Delbet drains to facilitate proper drainage and prevent fluid accumulation.

Postoperative care

The patient was sedated and intubated, and triple antibiotic therapy consisting of ceftriaxone, gentamicin, and metronidazole was continued. The day 1 postoperative care was marked by a fever of 38°C, hemodynamic stabilization under noradrenaline, and no abdominal distension. CRP level decreased to 30.9 mg/l, and prothrombin levels (28%) indicated disseminated intravascular coagulation (DIC) (Table 1). Blood culture results were negative. On day 2, the patient died of sepsis and multiorgan failure.

**Figure 4:**
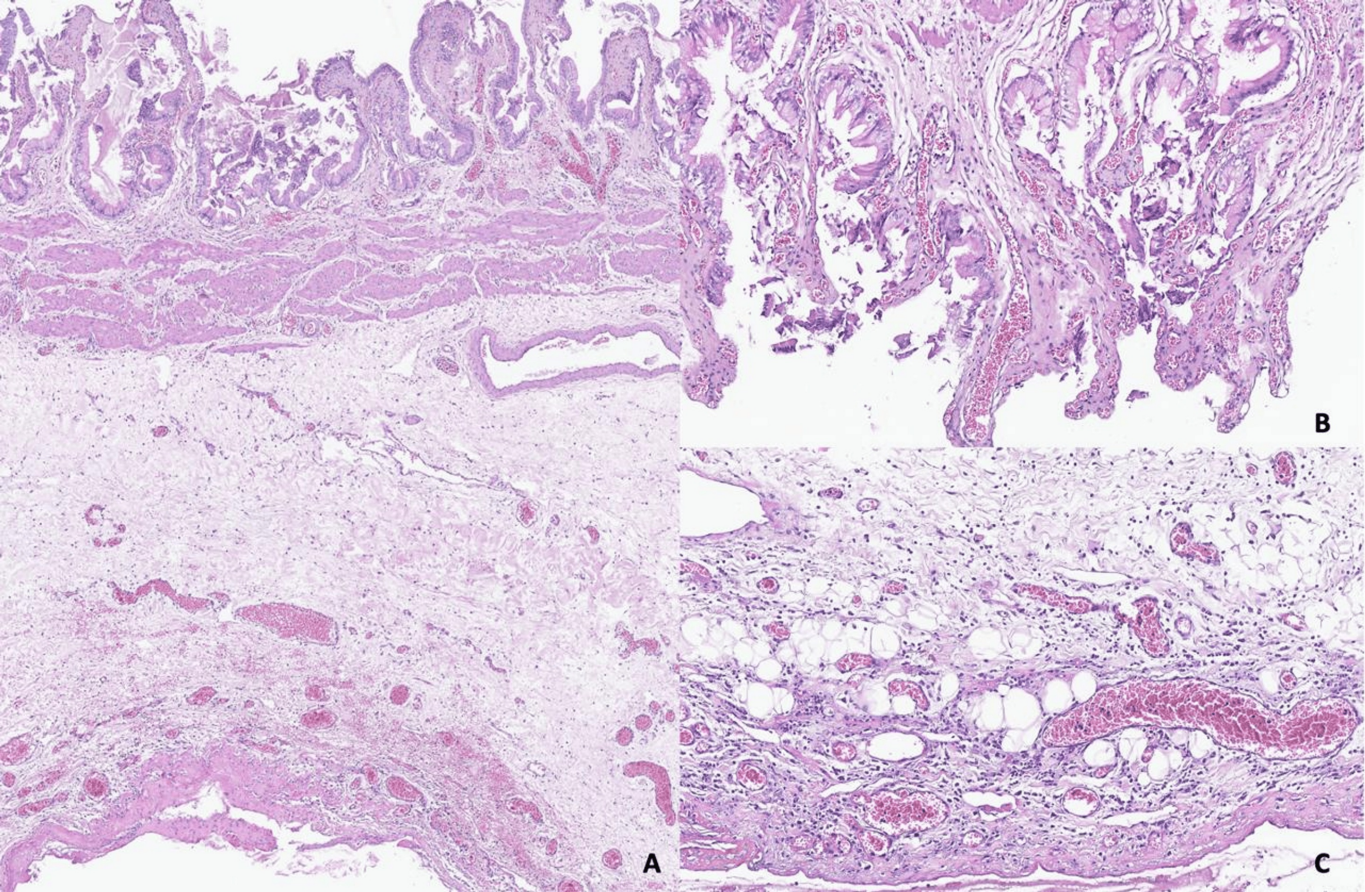
Representative micrographs of the gallbladder. Low-power view showing morphological features of subacute cholecystitis, with diffuse edematous, congestive, and inflammatory changes involving the entire thickness of the gallbladder wall (A). High-power view showing focal epithelial abrasion (B) with polymorphic inflammatory infiltrate extending to the subserosa (C) (hematoxylin and eosin; A: ×40, B-C: ×200).

Histopathological examination of the gallbladder revealed a slightly abraded epithelium in some areas, without any cytonuclear atypia. The underlying chorion was edematous and congestive, containing a diffuse, polymorphic inflammatory infiltrate composed of lymphocytes associated with neutrophils. The inflammatory infiltrate extended through all layers of the gallbladder wall. These morphological features are consistent with the diagnosis of subacute cholecystitis (Figure 4).

## Discussion

Duodenal ulcer perforation is an uncommon but well-recognized condition in children, with duodenal ulcers being more prevalent than gastric ulcers at a reported ratio of 18:4 [6]. While peptic ulcers are more frequent in adults [7], stress-related ulcers can occur in critically ill pediatric patients, particularly in cases of prolonged ICU stays. The incidence of stress-induced gastrointestinal ulcers in children ranges between 6% and 33% [8], and among those affected, 3-43% may develop upper gastrointestinal bleeding [9,10]. This can complicate the clinical picture and lead to significant morbidity. If not treated properly, it can lead to gastric or duodenal perforations, resulting in life-threatening conditions. Prophylactic methods to avoid stress-induced gastrointestinal complications may include PPIs such as omeprazole [10]. Early initiation of enteral feeding is crucial as it helps maintain splanchnic blood flow, potentially reducing the risk of stress-related mucosal damage [11].

Diagnosis of gastrointestinal perforations in sedated and intubated patients presents a unique challenge. Classical symptoms such as abdominal pain, guarding, and tenderness are often absent or obscured by the patient's critical condition and sedation. Thus, a delayed diagnosis can lead to a poorer prognosis. Radiological investigations, such as abdominal radiographs and CT scans, are crucial for diagnosing perforations because they can confirm pneumoperitoneum and localize the site of the perforation [12]. We were unable to perform this imaging owing to the patient's unstable condition. The sensitivity of plain abdominal X-rays for detecting organ perforations is generally lower than that of other imaging modalities, which explains our finding of no air leakage. This is because small amounts of free air or perforations in certain locations may not be visible on X-rays [13]. In this case, the decision to proceed with emergency laparotomy was based on clinical deterioration rather than radiological evidence, underscoring the importance of surgical judgment in complex ICU cases.

Gallbladder perforations are rare in children, and the main causes of these perforations include trauma, congenital anomalies, and infections [2,14,15]. The coexistence of the gallbladder and duodenal perforations is unusual. In adults, several reports have suggested that duodenal ulcer perforation may lead to localized inflammation and adhesions between the duodenum and gallbladder, predisposing the latter to secondary inflammatory damage and eventual perforation [16]. Konagaya et al. [3] reported a similar mechanism, in which inflammatory extension from a perforated duodenal ulcer induced acute cholecystitis and gallbladder wall breakdown due to direct serosal contact. We posit that the proximity of both perforations in our case supports the pathophysiological hypothesis that following duodenal perforation, the gallbladder wall was compromised by gastrointestinal enzymes in contact with it, resulting in gallbladder perforation.

To our knowledge, only one pediatric case of concurrent duodenal and gallbladder perforation has been previously documented, reported by Johnston in 1975 [5]. The primary cause was believed to be an extension of the erosive process of stress ulceration, which occurs because of severe illness, dehydration, and gastrointestinal distress. Despite the similarities with our case, Johnston described a case in which the diagnosis workup was facilitated by the patient’s consciousness and radiological findings, leading to sooner intervention and a good outcome. 

In our case, an exploratory laparotomy was urgently performed without confirming the cause of the onset because of the inability to perform a CT scan, and plain radiography was not useful. Despite the rarity of such occurrences, perforation of either one is a surgical emergency and can lead to septic peritonitis. In this case, the diagnosis of duodenal perforation was delayed because of its insidious onset. Surgical consultation was requested only after gastric bleeding occurred. Despite subsequent surgical intervention, the patient presented with advanced sepsis, which was ultimately fatal. Prompt recognition and intervention are critical to prevent further complications in patients hospitalized in the ICU because of the high risk of stress-induced ulcers. In this context, our patient was still sedated after the neurosurgical procedure, making it challenging to accurately assess her clinical status.

## Conclusions

Critically ill pediatric patients, especially those undergoing extended intensive care and sedation, are susceptible to stress ulcer pathologies, necessitating proactive prophylaxis, which may nonetheless prove inadequate. This case report documents the simultaneous occurrence of gastrointestinal and gallbladder perforation, emphasizing the potential pathophysiological connection between these conditions. Although exceedingly rare, this link warrants investigation if either condition is discovered perioperatively.
